# On the Action of Bromide of Potassium

**Published:** 1865-01

**Authors:** S. W. D. Williams

**Affiliations:** M. D., L. R. C. P., London


### ON THE ACTION OF BROMIDE OF POTASSIUM.

By S. W. D. WILLIAMS, M. D., L. R. C P, Lond.

Reading some remarks in a late number of the *Lancet* on the action of bromide of potassium, and having tried the drug extensively for the last five months, it has occurred to me that a few observations on its action may not be unacceptable to the readers of the *Medical Times and Gazette.*

Through the kindness of Dr. Wing, the Superintendent of the Northampton General Lunatic Asylum, I have been enabled freely to try it in as many as thirty-seven cases. These were all epileptics, and I append a table showing in one column the number of fits registered during the last five months of last year, when they were taking no medicine, and in the other the number registered during the first five months of this year, when each case was taking on an average ten grains of the salt twice daily.

I may premise that the greatest care was taken that, for the whole of the ten months during which these thirty-seven patients were under observation, their lives, with the exception of taking the bromide during the last five, should be spent under as near as possible the same circumstances.

| Males' Names. | Fits during the last five mos. of 1863. | Fits during the first five mos. of 1864. | Females' Names. | Fits during last five mos. of '63. | Fits during first five mos. of '64 |
|---|---|---|---|---|---|
| W. M. | 148 | 107 | E. H. | 23 | 19 |
| J. R. | 69 | 45 | E. J. | 25 | 37 |
| J. B. | 32 | 21 | M. K. | 60 | 27 |
| J. J. | 246 | 91 | E. H. | 29 | 9 |
| W. L. | 55 | 37 | E. W. | 50 | 56 |
| S. L. B. | 19 | 24 | C. S. | 17 | 23 |
| T. H. | 40 | 29 | S. A. | 82 | 85 |
| C. B. | 52 | 46 | M. L. | 20 | 5 |
| R. H. | 112 | 102 | A. S. | 41 | 22 |
| G. M. | 47 | 64 | E. G. | 46 | 53 |
| W. W. | 36 | 37 | H. W. | 1 | ... |
| J. L. M. | 33 | 26 | M. L. | 57 | 8 |
| T. G. | 13 | 4 | A. C. | 11 | 22 |
| R. G. | 30 | 9 | M. C. | 1 | ... |
| J. K. | 25 | 16 | S. A. P. | 577 | 556 |
| E. E. | 8 | 14 | S. A. | 1 | ... |
| W. O. | 10 | 10 | S. S. | 73 | 37 |
| W. M. | 29 | 14 | E. G. | 13 | 11 |
| J. J. | 8 | 10 | ..... ...... | .. | .. |
| | 1012 | 706 | | 1127 | 970 |

From the following table it will be seen that the number of fits amongst the males decreased by 306, and amongst the females by 157; that all the patients but 5 males and 6 females were benefited more or less; that the improvement was, however, more apparent amongst the males than the females; but that no patient of either sex was entirely cured. It is right to remark that all these patients are more or less insane, and many of them extremely violent at times.

Mr. Henry Behrend, the writer in the *Lancet*, confines his remarks to the powerful effect this drug has on "insomnia and restlessness, accompanied and dependent on nervous excitement and irritability," and this statement my own observations fully corroborate; but I have not the same confidence in recommending, as he does, the unfettered use of half-drachm doses; for in several of the cases recorded above it was found necessary to reduce even the average—ten grains twice daily; and in the majority the first use of the drug was accompanied by sickness and lassitude.

Those patients on whom the drug seemed to take the most effect in this way were seven in number; after using it for a few days the action of their hearts became slow and fluttering, the eye lost its lustre, the skin was cold and clammy; they had a wearied, anxious look, and complained of headache, and sickness, and shivering, and of unusual weakness at the knees, and invariably sat crouched up by the fireside all day, evidently devoid of all energy and resolution. Curiously enough, in all the cases thus powerfully affected the fits were increased instead of diminished.

The drug excited hypercatharsis in two patients, which was repeated again and again each time it was renewed; the fits in both these cases were diminished; in the case of the female from 41 to 22.

One patient, S. A., was apparently, five months ago, one of the most healthy persons in the home—fat, strong, and rosy; but soon after taking the bromide, the peculiar symptoms described above developed themselves, and the medicine was immediately omitted; but, although she rallied a little, her system never thoroughly recovered itself; tubercles became developed in the lungs, and she died towards the end of April. Truth compels me to confess that I have my doubts whether the bromide of potassium had not something to do with the poor girl's death—at all events, this occurrence has made me very watchful when using it.

On the other hand, considerable benefit has arisen from its use in some cases; it undoubtedly exercises a most powerful influence on the nervous system, and often soothes the irritability of epilepsy, even if it does not diminish the frequency of the fits, when no other medicine will take any effect, and in this way will be found a most valuable adjunct to the repertory of an asylum Dispensary. I cannot think that it has much effect, however, on the sexual system; for in some cases where it was used more especially with that view, there was no apparent result, but of its powers in inducing sleep in cases dependent on nervous irritability there can be no doubt, and often from ten to twenty grains twice daily will suffice to effect this.—*Med. Times and Gaz.*

